# Speckle disturbance limit in laser-based cinema projection systems

**DOI:** 10.1038/srep14105

**Published:** 2015-09-15

**Authors:** Guy Verschaffelt, Stijn Roelandt, Youri Meuret, Wendy Van den Broeck, Katriina Kilpi, Bram Lievens, An Jacobs, Peter Janssens, Hugo Thienpont

**Affiliations:** 1 Applied Physics research group (APHY), Vrije Universiteit Brussel, Pleinlaan 2, B-1050 Brussels, Belgium; 2 Brussels Photonics Team (B-PHOT), Vrije Universiteit Brussel, Pleinlaan 2, B-1050 Brussels, Belgium; 3 Light & Lighting Laboratory, KU Leuven, Gebroeders De Smetstraat 1, B-9000 Gent, Belgium; 4 iMinds research center for Studies on Media, Information and Telecommunication (SMIT), Vrije Universiteit Brussel, Pleinlaan 9, B-1050 Brussels, Belgium; 5 Barco - Projection Division, Noordlaan 5, B-8520 Kuurne, Belgium

## Abstract

In a multi-disciplinary effort, we investigate the level of speckle that can be tolerated in a laser cinema projector based on a quality of experience experiment with movie clips shown to a test audience in a real-life movie theatre setting. We identify a speckle disturbance threshold by statistically analyzing the observers’ responses for different values of the amount of speckle, which was monitored using a well-defined speckle measurement method. The analysis shows that the speckle perception of a human observer is not only dependent on the objectively measured amount of speckle, but it is also strongly influenced by the image content. The speckle disturbance limit for movies turns out to be substantially larger than that for still images, and hence is easier to attain.

One of the major contemporary challenges in cinema projection is the further increase of the amount of light, expressed in lumens, projected on the screen. Due to the limitations of arc lamps^1^ currently used in high-end projectors, the luminance or brightness of projected cinema images is considered by many people as being too dark^2^, especially for 3D cinema projection where a considerable amount of light is lost because of the filtering involved in creating separate views for each eye. Moreover, the output of the arc lamp will drop considerably during its lifetime^3^, which itself is limited to a few hundreds of hours^4^ leading to substantial replacement costs when operating such arc-lamp based projectors.

Laser display technology is widely considered as the most promising technology for high-lumen future projection applications^2^. These sources can combine a high lumen output with a very low étendue, which leads to a high optical efficiency in the projector^5^. The lifetime of a laser is typically one order of magnitude larger than that of arc lamps, and the output power does not degrade strongly over the laser’s lifetime^6^. Laser projectors can therefore potentially outperform current arc lamp projectors in many different aspects.

The introduction of lasers in projection systems has been hampered by the presence of speckle in projected images^7^. This speckle pattern is a granular pattern of spots which is overlaid on the projected image. Speckle arises due to the quasi-random interference that is generated because the coherent laser beam is scattered from a projection screen that is rough on the scale of the optical wavelength. As the speckle pattern in a laser projector is usually the sum of multiple, independent speckle patterns each of which is created by a large number of independent scattering elements, the intensity of the speckle pattern typically obeys Gaussian statistics (see^8^, and supplementary data 1). The amount of speckle is usually quantified by the speckle contrast value C = sigma/mean, where the numerator is the standard deviation of the intensity fluctuations and the denominator is the mean intensity.

The speckle pattern is displeasing for a human observer and therefore it needs to be reduced to an acceptable level. There exist several techniques that can be used to reduce the amount of speckle^9^,^10^,^11^. These techniques are based on the superposition of (partly) uncorrelated speckle patterns, which are usually generated using wavelength decorrelation, spatial decorrelation, angular decorrelation, screen movement, polarization scrambling or any combination thereof^7^. When N independent speckle patterns with equal average intensity are overlapped on the screen, the speckle contrast will be reduced with a factor 1/sqrt(N). The speckle contrast can thus always be further reduced by combining a larger amount of independent speckle patterns, but this will usually require a more complex, and hence a more costly, projection system. For the future development of new laser based projection systems it is therefore important to know the highest amount of speckle that can be tolerated in projected images before the speckle becomes disturbing for a human observer.

When quantifying the speckle disturbance limit, it is also important to take into account the method that has been used to objectively measure the speckle contrast. The speckle contrast is usually quantified by measuring the intensity distribution of a uniform image captured by a CCD camera. The measured speckle contrast will then depend on the settings of the camera system, such as the camera’s pixel size, focal length, f-number and integration time. In order to be able to compare speckle contrast values from different studies and/or from different projectors, it is imperative to disclose all details of the speckle measurement procedure and the value of each of the measurement parameters.

Previous studies^3^,^12^,^13^ resulted in an estimate of the speckle detection threshold for still images of about 3%, but these reports do not fully describe the speckle measurement procedure that was employed. We have studied^14^ the speckle detection limit for still images, where we quantified the speckle contrast using the well-defined measurement procedure outlined in previous work^15^ and which is based on the speckle perception of a human observer. From this quality-of-experience subjective experiment, incorporating the responses of 40 test persons, we estimate the speckle detection limit for still images to be 3.6% for red (639 nm), 3.2% for green (532 nm) and 4.4% for the blue (465 nm) primary colors of the projector.

For moving images, however, hardly any research has been done on the level of speckle that can be allowed before a human observer finds it disturbing. It can be expected that the speckle disturbance limit for moving images might be different from that for still images. Therefore, the speckle disturbance limit for moving images still needs to be identified. Quantifying this subjective human perception of speckle is not easy, and requires a multi-disciplinary approach in which social research methodologies are combined with quantitative measurements of speckle patterns.

In this paper we investigate the speckle perception for moving images in a cinema environment. For this purpose, we equipped a movie theatre room with a prototype laser projector. A group of 186 participants was gathered to evaluate the speckle perception of the moving images in a subjective ‘Quality of Experience’ (QoE) experiment^16^. The speckle disturbance limit is assessed using several, short movie trailers, and the speckle contrast value of each trailer is evaluated using the measurement procedure outlined in^15^. Finally, the speckle disturbance of a laser-projected full-length film is evaluated when the participants are unaware that they are still participating in a QoE experiment with the laser-projector. This way speckle disturbance can be measured in a realistic cinema setting in which people watch a full-movie and are not specifically focusing on image quality or speckle perception.

## Results

### Setup of the projection system

**Figure 1:**
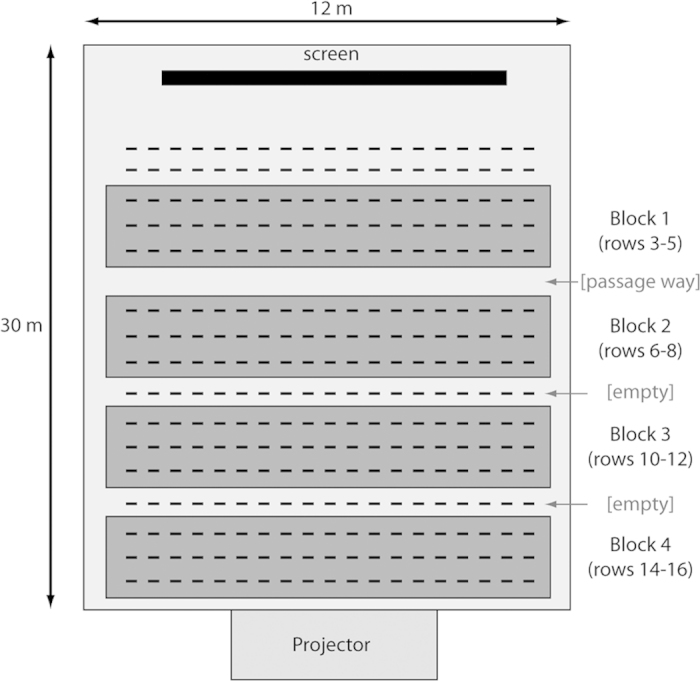
Dimensions and divisions of the cinema theatre room used during the speckle perceptions tests. At the right, we indicate how the responses of people seated in different rows are regrouped in blocks for the statistical analysis.

The laser projection system is placed in the control room at the back of a cinema theatre room. The theatre room is about 30 m long and has a projection screen with a width of 10 m. The projector has a resolution of 4096 × 2160 pixels and its white point is set at the CIE chromaticity diagram coordinates (0.314, 0.351). The luminance of the projected images is set at 48 NIT, and the wavelength of the primary colors is 463 nm for blue, 532 nm for green and 634 nm for red. A schematic layout of the room is given in Fig. 1. The projection system uses, amongst others, angular decorrelation as a means to reduce speckle. The effect of angular decorrelation is linearly depending on the angular extent of the observer’s entrance pupil relative to the screen^7^. This implies that the observed speckle contrast will be smaller if the distance from the observer to the screen increases. Therefore, we will group the participants into 4 different blocks when we perform statistical analysis of the responses. This grouping is illustrated in Fig. 1: Block 1 corresponds to all the participants seated in rows 3 to 5, Block 2 is the collection of all persons seated in rows 6 to 8, etc. Additionally, a polarizer can be placed after the projection lens to further increase the speckle contrast. The polarizer reduces the brightness of the images on the screen, so when no polarizer is used, a neutral density (ND) filter can be used to reduce the brightness of the images (without influencing the speckle contrast) to the same level as when placing a polarizer in the beam’s path.

**Figure 2:**
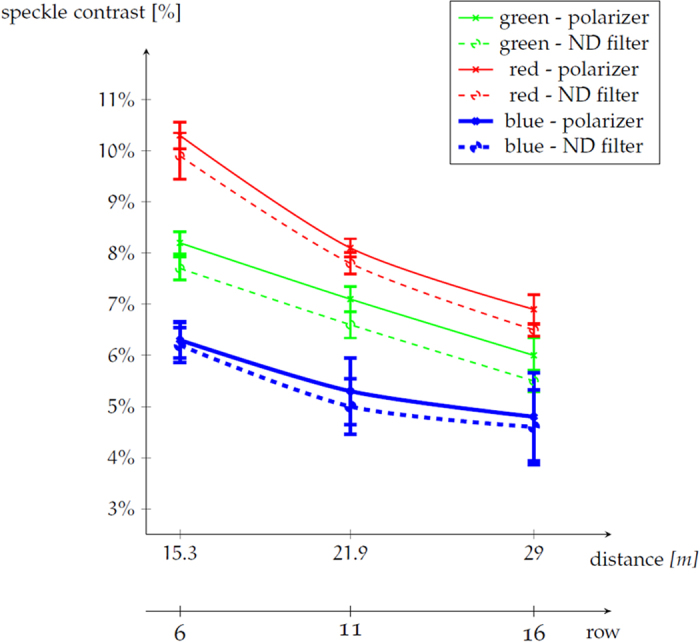
The speckle contrast values of the three primary colors of the laser projector as a function of the distance from the screen in the cinema theatre room. At the bottom we also indicate the position of different rows. Each speckle contrast is the average of about 20 measurements. The symbols are the actual averaged measurement points, the lines are included to guide the eye. The solid lines correspond to the speckle contrast values when a polarizer is placed in front of the projection system and the dashed lines represent the speckle contrast values when a neutral density filter is placed in front of the projection system. Vertical bars correspond to the standard deviation of the measured speckle contrast values.

The speckle contrast values are measured from static images with a uniform intensity distribution using the measurement procedure outlined in the Methods section^15^. The resulting speckle contrast values for the three primary colors (both with polarizer and neutral density filter) are depicted in Fig. 2 as a function of the row, where larger row numbers are located further away from the screen. Note that we have no measurement of the speckle contrast at the first rows of the room, because the measured intensity distribution at these short distances still contained fluctuations due to the finite size of the projector’s pixels on the screen. At larger distances, these pixels are no longer resolved on the CCD camera. Also note that the speckle contrast indeed decreases approximately linearly with increasing viewing distance because of angular decorrelation The speckle contrast decreases slightly when no polarizer is placed in the beam path. We will use Fig. 2 in order to link the user responses - grouped in the different blocks according to Fig. 1- with an objectively measured amount of speckle.

**Figure 3:**
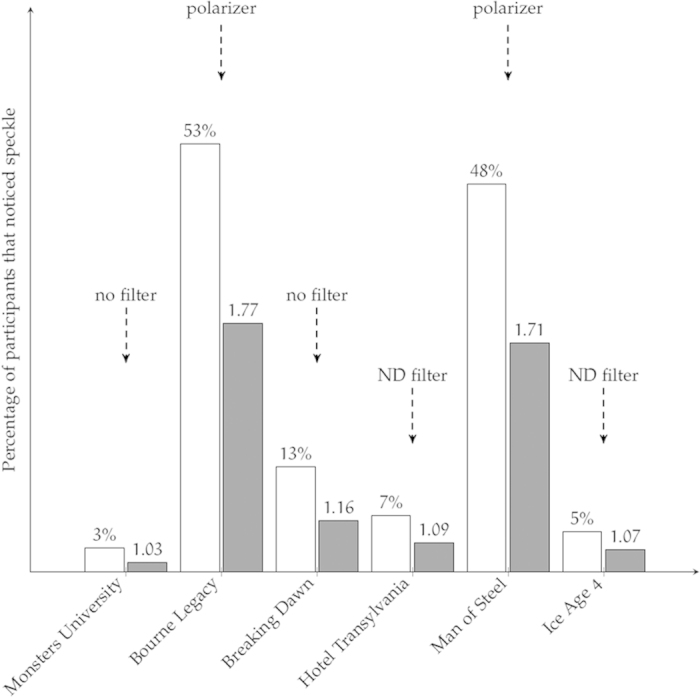
Percentage of the people that observed speckle in the different movie trailers (white bars), together with the speckle Mean Opinion Score (MOS) (grey bars) for each trailer. The text at the top of each column indicates the projector setting for the different trailers.

A total of 186 persons participated in the test, which were almost evenly distributed over the different blocks in the room. In an initial phase of the test, speckle was explained to the participants using some images in which we artificially embedded a speckle-like granularity in the image’s intensity distribution. To make respondents familiar with the used voting system for the test, some simple questions were asked (e.g. how did you get here, gender, color blindness). Afterwards, six different trailers are shown, three trailers of animated movies (*Monsters University, Hotel Transsylvania, Ice Age 4)* and three trailers of regular movies with different content (*Man of Steel, Bourne Legacy, Twilight Saga: Breaking Dawn)*. The trailers are shown with a polarizer, a neutral density filter or neither as is specified at the top of Fig. 3. After every trailer, the participants are asked to evaluate the speckle quality with a score ranging from 1 (imperceptible) to 5 (very disturbing). The exact grading of the scores is shown in Table 1. After the trailers, the participants are shown a full-length movie called *TED* while they are unaware of the fact that the movie is shown using a laser projector and that this movie is still part of the experiment. The movie is presented as a reward for participating in the test and provides respondents with a realistic cinema experience. After the full-length movie, the participants are again asked to evaluate the amount of speckle using the same scale as before. Finally, focus group interviews were taken from 35 participants seated at different locations in the theatre room. This allows the participants to motivate some of their answers and provide an in-depth discussion on the possibility of other parameters influencing their judgment.

### Influence of projector settings and movie content

For every movie trailer, the corresponding percentage of people that noticed speckle is shown in Fig. 3. In this figure, we also show the Mean Opinion Score (MOS) for each trailer on the scale expressed in Table 1. Interestingly, there are four trailers for which the majority of the people did not notice speckle. Only for the trailers of the movies *Bourne Legacy* and *Man of Steel*, about half of the people noticed speckle and the speckle MOS is significantly higher than what we expect to find for a lamp-based projector. We want to remark that we did not find significant differences in the speckle perception when considering age or gender.

From Fig. 3 it is clear that the movie content exerts an influence on the perception of speckle. The percentage of people that noticed speckle in the *Monsters University* trailer is about 4 times lower than in the *Breaking Dawn* trailer. Nevertheless, both trailers are shown with exactly the same projector settings, resulting in identical amounts of speckle in the projected images. The speckle MOS is typically higher for those trailers that contain large grey areas (e.g. from a wall or a cloudy sky) with a rather uniform intensity distribution and that change slowly in time. The small difference in the measured speckle contrast with and without polarizer (see Fig. 2) indicates that the high percentage of people noticing speckle in the *Bourne Legacy* and *Man of Steel* trailers is mainly due to the content and not so much due to the polarizer.

### Speckle disturbance limit

We further investigate the speckle disturbance limit by evaluating the responses concerning the *Man of Steel* trailer as there is a considerable amount of people that noticed speckle in this trailer. Remark that we do not further study in detail the results of the *Bourne Legacy* trailer, as this trailer contained some special effects that might have been confused by the audience with speckle.

There is no clear threshold for the observation of speckle: some people will observe speckle even when the speckle contrast is very low, while other people do not see it at high speckle contrast values. Therefore, we first have to decide on a useful threshold that tells us when speckle becomes too large. We have chosen in this paper to set this threshold based on the perception of speckle in still images using a lamp projector that we know from a previous study^14^: when we asked a test audience to evaluate the speckle perception of still images in a lamp projector, this resulted in a speckle MOS of 1.5 on the scale defined by Table 1. The objectively measured speckle contrast of this lamp projector is close to zero, but the audience in the test^14^ did not know when they were evaluating a laser projector and when they were looking at images projected with a lamp projector. Therefore, if the speckle MOS of the laser projector is below 1.5, this indicates that people perceive the same amount of speckle as for a lamp projector, and there is no need to try to further lower the speckle MOS below 1.5. For that reason we set the speckle disturbance limit as the objectively measured speckle contrast at which a speckle MOS of 1.5 is reached.

The distribution of the observers score’s for the speckle perception is not Gaussian (see supplementary data 2). Therefore, we use a non-parametric Kruskal-Wallis test^17^ to identify statistically significant differences between the responses of the people sitting in different blocks. The results of this analysis are summarized in Table 2, in which we show the number of responses N, the speckle MOS for each block and the mean difference in speckle scores between the blocks. From this table, it can be deduced that the speckle MOS decreases as people are seated further away from the screen. This reduction of the speckle MOS is due to the decrease in the amount of speckle observed in Fig. 2 when the distance to the screen is increased. It is important to note that the speckle MOS is slightly below 1.5 for the last block, i.e. for Block 4. The position corresponding to the speckle disturbance limit can thus be located between Block 3 and Block 4.

Looking at the mean difference in speckle score, it is clear that Block 4 is statistically significantly different from all the other blocks. This implies that there is a clear difference in speckle perception between the third and the fourth block. The speckle MOS decreases from 1.74 to 1.41. As we aim to achieve a speckle MOS smaller than 1.5 (because this is equivalent to the speckle perception in a lamp projector), the speckle disturbance limit will be close to the speckle contrast reported in Fig. 2 for row 16 with polarizer, i.e. the speckle disturbance limit for moving images is reached in our setup for C = (6.9 ± 0.3)% for red, C = (6.0 ± 0.3)% for green, and C = (4.8 ± 0.9)% for blue.

Additionally, it is interesting to investigate a movie trailer where only few people noticed speckle and verify whether or not there is an influence of the row number on the speckle perception. We use the trailer of the movie *Monsters University* as an example where almost nobody noticed speckle (3%). We again perform a non-parametric Kruskal-Wallis test in order to identify statistically significant differences in the responses of the people sitting in different blocks (see Table 3). The speckle MOS of the different blocks is close to 1, meaning that almost everyone evaluates the speckle as being imperceptible. Furthermore, there is no significant difference between the MOS scores of the different blocks.

## Discussion

After the speckle perception tests with the movie trailers, the participants were rewarded by showing them the movie *TED*. What they did not know was that the movie itself was projected using a laser projector. Furthermore, the speckle settings were different before and after the break. Before the break, a polarizer was placed in front of the projection system, while after the break, the neutral density filter was used. As the participants were not aware of the additional experiment, their primary focus was on the content, rather than on identifying speckle.

After the movie, the participants were asked whether they noticed speckle in the movie. A majority of the people mentioned that speckle was imperceptible (69.4%), followed by 29.0% of the participants mentioning that speckle was perceptible, but not annoying. The remaining 1.6% rates the speckle quality slightly annoying to very annoying. That means that 98.4% of the participants was watching a movie –projected by a prototype laser projector– and was not disturbed by the fact that there was (a relatively high amount of) speckle in the images (with a speckle contrast up to 8–10% depending on the color and the position in the room). The speckle MOS value –which was equal to 1.33– is slightly dependent on the position, but this dependency is not statistically significant. This result indicates that the speckle perception of the *Man Of Steel*-trailer probably is the worst case scenario.

Furthermore, when the participants were asked whether they noticed a difference in the amount of speckle before and after the break, 88.6% did not notice a difference. For the remaining part, 5.8% preferred the image quality before the break (with the polarizer and thus higher speckle) and the remaining 5.3% preferred the image quality after the break. Therefore, there was no significant difference in the quality perception with and without the polarizer and the corresponding small difference in speckle contrast (see Fig. 2) is insufficient to be noticed.

The speckle disturbance limit in the case of moving images turns out to be much higher than for still images. In previous work^14^ we used a similar procedure as the one described in this paper to estimate the speckle perception of still images, leading to a speckle disturbance limit being equal to the speckle detection limit of 3.6% for the red (639 nm), C = 3.2% for the green (532 nm) and C = 4.4% for the blue (465 nm) primary colors. In the study presented here using moving images, we could not independently change the speckle contrast of each of the primary colors. Therefore, it is possible that the measured speckle disturbance limit is mainly dominated by the speckle disturbance caused by one of the primary colors, and that the speckle disturbance limit of the other colors is actually (slightly) larger. As the speckle contrast is always largest for the red primary color in our projector setup, it is thus probable that the disturbance limit is set by the speckle at the red wavelength channel. We find that, for moving images, the speckle becomes disturbing if the speckle contrast becomes larger than (6.9 ± 0.3) for the red, (6.0 ± 0.3)% for the green, and (4.8 ± 0.9)% for the blue primary colors of the projector.

## Methods

### Speckle measurement procedure

The measurement setup used to objectively quantify the amount of speckle is based on the findings presented in literature^15^. The measurement setup consists of a camera zoom-lens (Nikon Nikkor AF-D 24–85 mm) mounted on a 12-bit monochrome CCD camera (Ophir Spiricon SP620U) with a pixel area of 4.40 × 4.40 μm^2^. In order to objectively measure the speckle contrast and define a human speckle disturbance limit, one should make sure the speckle contrast measurement is independent of the projection system and is related to the perception of speckle by a human observer. As a consequence, the camera setting should exhibit a clear aperture of 3.2mm and a square root ratio between the pixel area Ap and the speckle area Ac of Sqrt(Ap/Ac) = 0.54. These values are based on the typical characteristics of the human eye, and they lead to a pixel size that is small enough such that this pixel size has a negligible effect on the measured speckle contrast^15^. For the pixel area of the used CCD camera, this corresponds to a focal length of the camera lens of 43.4 mm and an f-number of 13.4. As we can only set the f-number of the used camera lens in discrete steps, we set the f-number to 16, which is the lens’s setting closest to and higher than the desired value. We adjust the focal length to 51 mm such that the clear aperture of the camera lens remains at 3.2 mm (being equal to the clear aperture of the human eye at a luminance of 48 NIT^18^). These settings of the f-number and the focal length result in a ratio Sqrt(Ap/Ac) = 0.46, which is only slightly smaller than the desired value of 0.54 and which leads to a negligible change in the speckle averaging due to the finite pixel size^15^. We make sure that the screen is sharply imaged on the CCD of the camera. Images are acquired in a cinema room with the ambient lighting switched off, resulting in a background ambient light level of about 0.5 NIT. The integration time of the camera is set to 400 ms, which is larger than the 50 ms integration time we proposed in^15^. This increase in the integration time was needed in order to reduce the noise to an acceptable level, while it should not influence the measured speckle contrast as the laser projector does not use time averaging of a moving speckle pattern as one of the methods to reduce speckle. The electrical camera noise is taken into account by subtracting the (intensity weighted) camera noise from the measured standard deviation of the intensity distribution (see supplementary method 1). Finally, small fluctuations in the intensity across the screen (e.g. due to screen non-uniformities) are typically present at low-spatial frequencies and are removed from the captured images by high-pass spatial filtering (see supplementary method 2).

### Human perception test procedure

The applied test procedure for the subjective QoE experiment is based on the standardized procedures for subjective audiovisual quality assessment as defined by International Telecommunication Union (ITU)-T Recommendation P.910^19^ and ITU-R Recommendation BT.500-12^20^. These recommendations provide guidelines related to the number of users to include (min. 15), the physical setting of the test (e.g. allowed room illumination, display brightness and contrast, and viewing distance between the viewer and the screen) and the trail structures for the test. Results of these QoE experiments are often applied to calculate the effects of video encoding and transmission on end users’ perceived quality. These experiments are typically conducted in a lab setting, although recently also experiments within the natural context of the use case are being conducted, taking into account the contextual factors that influence the viewing experience^15^. In these experiments, focus is shifted from the evaluation of visual quality in short fragments, to the more natural experience of watching a full movie (see supplementary discussion)^21^.

## Additional Information

**How to cite this article**: Verschaffelt, G. *et al.* Speckle disturbance limit in laser-based cinema projection systems. *Sci. Rep.* **5**, 14105; doi: 10.1038/srep14105 (2015).

## Supplementary Material

Supplementary Information

## Figures and Tables

**Table 1:** Table showing the grading of the scores for speckle perception in the questionnaire.

Mark for the images how you see speckle	
Imperceptible	1
Perceptible, but not annoying	2
Slightly annoying	3
Annoying	4
Very annoying	5

**Table 2:** Analysis of speckle Mean Opinion Score (MOS) and mean difference in speckle score of the *Man of Steel* trailer, and how these quantities depend on the block number.

	N	Speckle MOS	Mean difference speckle score			
			Block 1	Block 2	Block 3	Block 4
Block 1	46	1.98	—	−0.24	−0.23	−0.57**
Block 2	42	1.74		—	0.01	−0.33*
Block 3	43	1.74			—	−0.33**
Block 4	51	1.41				—

N is the total number of observations within each data set. ^*^indicates differences that are statistically significant at p = 0.05.

^**^indicates differences that are statistically significant at p = 0.01.

**Table 3:** Analysis of speckle Mean Opinion Score (MOS) and mean difference in speckle score of the *Monsters University* trailer, and how these quantities depend on the block number.

	N	Speckle MOS	Mean difference speckle score			
			Block 1	Block 2	Block 3	Block 4
Block 1	46	1.02	—	0.03	0.00	0.02
Block 2	42	1.05		—	−0.03	−0.01
Block 3	47	1.02			—	0.02
Block 4	51	1.04				—

N is the total number of observations within each data set. *indicates differences that are statistically significant at p = 0.05.

**indicates differences that are statistically significant at p = 0.01.
