# Supplementary material for: Speckle disturbance limit in laser-based cinema projection systems
Source: Sci Rep. 2015 Sep 15;5:14105. doi: 10.1038/srep14105 (PMC4572931; doi:10.1038/srep14105)
Supplement: Supplementary Information [file srep14105-s1.pdf]

Speckle disturbance limit in laser-based cinema projection systems

Guy Verschaffelt[1,*], Stijn Roelandt[2], Youri Meuret[2,3], Wendy Van den Broeck[4], Katriina Kilpi[4], Bram Lievens[4], An Jacobs[4], Peter Janssens[5], Hugo Thienpont[2]

[1] Applied Physics research group (APHY), Vrije Universiteit Brussel, Pleinlaan 2, B-1050 Brussels, Belgium,

[2] Brussels Photonics Team (B-PHOT), Vrije Universiteit Brussel, Pleinlaan 2, B-1050 Brussels, Belgium,

[3] Light & Lighting Laboratory, KU Leuven, Gebroeders De Smetstraat 1, B-9000 Gent, Belgium,

[4] iMinds research center for Studies on Media, Information and Telecommunication (SMIT), Vrije Universiteit Brussel, Pleinlaan 9, B-1050 Brussels, Belgium,

[5] Barco - Projection Division, Noordlaan 5, B-8520 Kuurne, Belgium.

Supplementary material

Supplementary Method 1: Calibration of electrical camera noise. The amount of speckle noise is extracted from measurements with a CCD camera of the intensity distribution across the screen by measuring the average intensity $I_{measured}$ and the intensity's standard deviation $\sigma_{measured}$ of the captured image. This leads to a measured speckle contrast $C_{measured}=\sigma_{measured} / I_{measured}$. The measurement of $\sigma_{measured}$ contains a contribution from the speckle pattern and a contribution from the electrical noise of the camera. Under the assumption that both phenomena lead to independent Gaussian intensity distributions, we can subtract the influence of the CCD noise. The CCD noise is characterized by taking a measurement of the intensity distribution while the camera lens is blocked for any incoming light. The resulting intensity distribution has an average very close to zero, and a standard deviation σ_{CCD}. The actual value of the speckle contrast C is then given by

$$C = \sqrt{C_{measured}^2 - \frac{\sigma_{CCD}^2}{I_{measured}^2}}$$

This subtraction of the CCD noise is done for each measurement of the speckle contrast reported in the manuscript.

Supplementary Method 2: High-pass spatial filtering and absence of the projector's pixels in captured speckle images. Most of the captured images contain some fluctuations of the intensity distribution on a large scale, i.e. at small spatial frequencies, due to non-homogeneities in the screen and in the projector. In order to remove these slow fluctuations, such that they do not influence the measured speckle contrast, we first subject the image to a low-pass mean spatial filter. The originally measured intensity distribution is then divided by the filtered one. This results in a new distribution from which slow variations of the intensity are removed. The low-pass spatial filtering is performed by calculating the spatial convolution between the image and a kernel. We used a normalized circular kernel with radius N_{pixels}. Such a filter smoothens the image by replacing each pixel intensity by the neighborhood mean intensity. The size of the kernel N_{pixels} should not be too small because then the speckle spots will be (partially) filtered out, nor should it be too large, because then the filtering will not remove the background intensity fluctuations. We take N_{pixels} = 25, which is large compared to the speckle spots in the captured images. We verified that we consistently obtain a similar speckle contrast if we change the kernel slightly from 20 to 50 pixels. Examples of the original image, the image after low-pass filtering, and the final image from which the speckle contrast is estimated, are given below in Figures A, B and C. The original image was captured in the middle of the room (row 11), while the screen was illuminated by a uniform image with only the red primary color switched on. The speckle contrast $C_{measured}$ is obtained from Fig. C by manually selecting several regions-of-interest (ROI) that do not contain a dark spot caused by dust particles on the CCD. Remark that the speckle contrast is normalized with respect to the average intensity. Therefore we can measure the speckle contrast $C_{measured}$ in each ROI from Fig. C instead of using the original CCD image given in Fig. A. The corresponding mean intensity $I_{measured}$ in each ROI is extracted from Fig. A. The measurements of the speckle contrast are then corrected for electrical camera noise, yielding a value of the speckle contrast C in each ROI. The average of these values is used in Fig. 2 of the manuscript, while the standard deviation of the speckle contrasts from the different ROIs is used in Fig. 2 in order to indicate the measurement errors. Finally, we show in Fig. D a transverse profile along the line indicated in Fig. C, showing that the average intensity in Fig. C is close to 1.

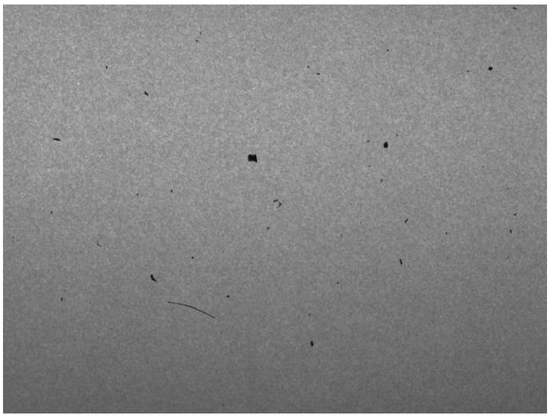

Figure A: Original image captured by the CCD camera, when the screen is illuminated by a uniform red image. Only part of the screen is captured by the CCD camera. The black spots in the image are due to dust particles that cover some pixels of the CCD.

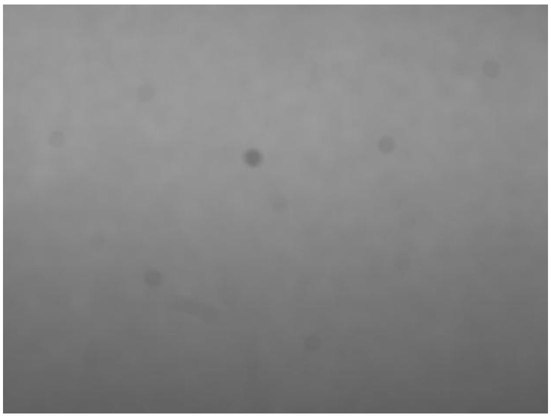

Figure B: Image after low-pass filtering of Figure A, using a kernel with radius N_{pixels} = 25.

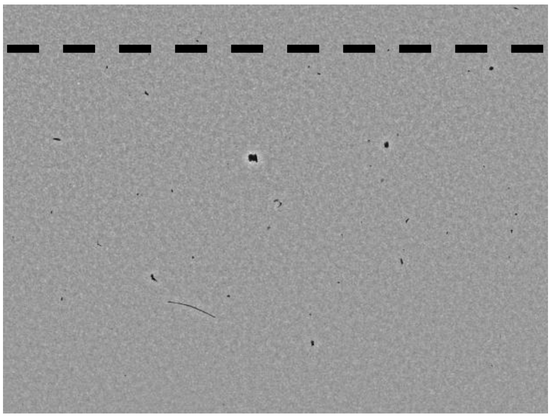

Figure C: Final image obtained by dividing Figure A pixel-by-pixel with Figure B. Multiple measurements of the speckle contrast $C_{measured}$ are obtained from this figure by selecting several regions-of-interest that do not contain a dark spot caused by dust particles on the CCD.

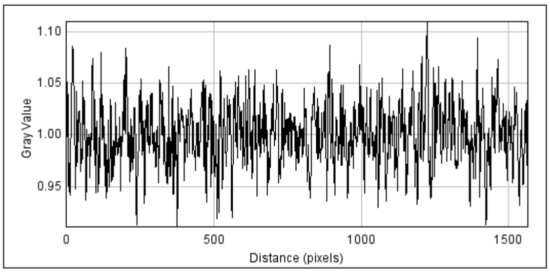

Figure D: Transverse profile of the intensity along the line indicated in Fig. C, showing that the average intensity in Fig. C is close to 1.

The speckle contrast measurement can contain an additional measurement error if the projector's pixels are resolved in the image captured by the CCD camera. The visibility of the pixels' edges can lead to small intensity variations, which increase the standard deviation extracted from the captured image. Typically, these pixels should not be visible as our speckle measurement procedure is linked

to the characteristics of the human eye, and the projector's pixel size will be designed such that they are not seen by a human observer. We check that these pixels are not present in the captured image distributions by calculating the spatial FFT of the filtered image. Remnants of the projector's pixels will be visible as sharp spots at a well-defined spatial frequency. Such spots are not visible in Fig. E, which is the spatial FFT of (part of) Fig. C. For comparison, we also show the FFT of a speckle pattern measured with a higher spatial resolution (see Fig. F) where these spots at the inverse of the pixel size are clearly identifiable.

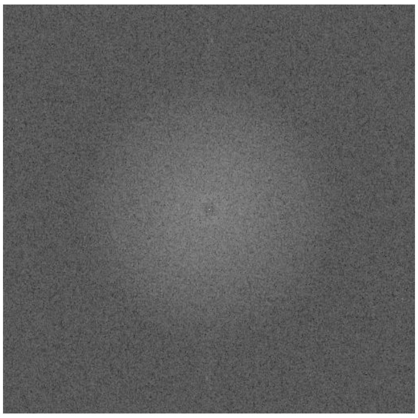

Figure E: FFT of (part of) Figure C, where the horizontal and vertical edges correspond to a spatial frequency of 0.5 cycles per pixel

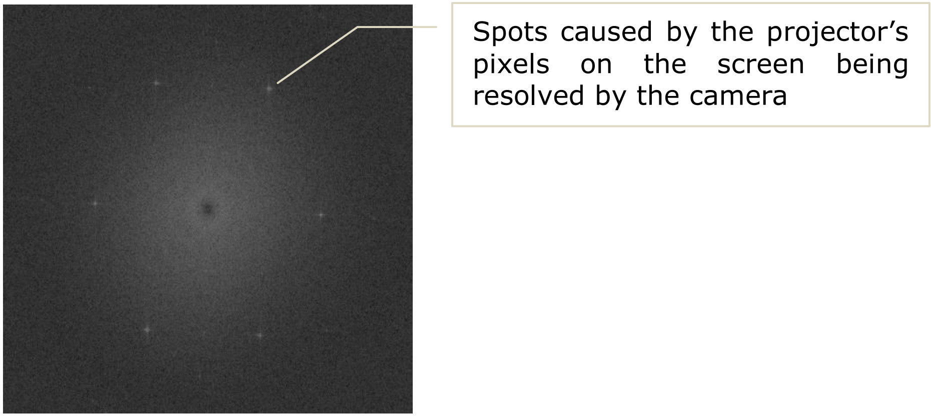

Figure F: FFT of a speckle pattern that was recorded with a higher spatial resolution, such that the projector's pixels show up as bright spots.

Supplementary Data 1: Gaussian intensity distribution of measured speckle patterns. The intensity of a speckle pattern will have a negative exponential distribution (with a speckle contrast equal to 1) if the light scattered from the different scattering elements is fully coherent. However, this is typically not the case in a laser projector, where techniques will be used to lower the speckle contrast. These techniques are based on the superposition of (partly) uncorrelated speckle patterns. If a large number of these uncorrelated patterns are summed on an intensity basis, then the intensity of this superposition will have a (approximately) Gaussian distribution. In our projector, we observe speckle contrasts of 0.1 or lower. As the speckle contrast (for N independent patterns with equal intensity) is given by 1/sqrt(N), the number of independent patterns in our setup is larger than 100, and we will observe Gaussian statistics in

the intensity. An example of such a Gaussian intensity distribution is shown in Fig. G, where we plot a histogram of the intensity of the speckle pattern shown in Fig. C (after exclusion of the regions in Fig. C containing dust particles).

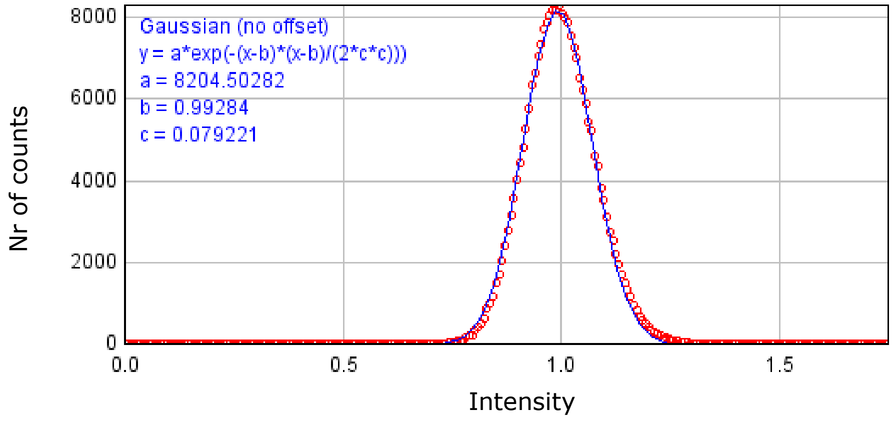

Figure G: Histogram of the intensity of the speckle pattern shown in Fig. C (red dots), and fit to a Gaussian function (blue line) illustrating that we typically observe Gaussian statistics.

Supplementary Data 2: Non-Gaussian distribution of user responses. In Fig. H, the speckle scores of all user responses are plotted when the laser projector is used to show the *Bourne Legacy* trailer. The solid curve is a normal distribution fit based on the mean opinion score (MOS) and the standard deviation of the observer scores. This curve does not match well with the histogram of the observer scores. Similarly shaped distributions of the observer scores are also found for the other trailers. Therefore, we have to make use of a non-parametric statistical test in order to analyze the data.

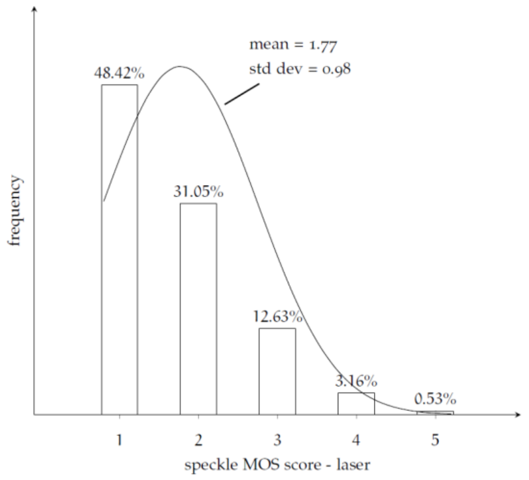

Figure H: Histogram of the speckle score of all user responses when the laser projector is used to show the *Bourne Legacy* trailer.

Supplementary Discussion: Contextual factors in human perception test procedures. In the specific test procedure of this large-scale QoE experiment in the movie theatre, we combined two approaches. In the first part of the test set-

up, we applied a standardized approach in which users are evaluating shorter video fragments following the guidelines mentioned in the manuscript. Users are aware that they are participating in a QoE experiment and specifically focus on the quality (in this case speckle perception). The selected setting is a realistic setting (movie theatre), and the number of subjects (N=186) is also much higher than the prescribed N. The high number of respondents allowed us to differentiate amongst user profiles (as we also gathered detailed profile information of all respondents) and in seating position in the movie theatre. As respondents were asked to bring their friends and family, this also led to a more natural experience, which was very important for the second part of the test. Here, a blind QoE experiment was conducted in which respondents watch a full movie as they would normally do in a movie theatre, including a box of popcorn and a soft drink. This way, users are focused on the narratives of the movie and are in the flow of the movie. This allows us to investigate differences in speckle perception when respondents are focused on quality evaluation and when they are focused on the content as is normally the case in a movie theatre setting.
